# Use of Functional Linear Models to Detect Associations between Characteristics of Walking and Continuous Responses Using Accelerometry Data

**DOI:** 10.3390/s20216394

**Published:** 2020-11-09

**Authors:** William F. Fadel, Jacek K. Urbanek, Nancy W. Glynn, Jaroslaw Harezlak

**Affiliations:** 1 Department of Biostatistics, Fairbanks School of Public Health, Indiana University, Indianapolis, IN 46202, USA; 2 Department of Medicine, Division of Geriatric Medicine and Gerontology, School of Medicine, Johns Hopkins University, Baltimore, MD 21205, USA; jurbane2@jhu.edu; 3 Department of Epidemiology, Graduate School of Public Health, University of Pittsburgh, Pittsburgh, PA 15261, USA; epidnwg@pitt.edu; 4 Department of Epidemiology and Biostatistics, Indiana University, Bloomington, IN 47405, USA

**Keywords:** accelerometry, physical activity, Fourier transform, functional linear model

## Abstract

Various methods exist to measure physical activity. Subjective methods, such as diaries and surveys, are relatively inexpensive ways of measuring one’s physical activity; however, they are prone to measurement error and bias due to self-reporting. Wearable accelerometers offer a non-invasive and objective measure of one’s physical activity and are now widely used in observational studies. Accelerometers record high frequency data and each produce an unlabeled time series at the sub-second level. An important activity to identify from the data collected is walking, since it is often the only form of activity for certain populations. Currently, most methods use an activity summary which ignores the nuances of walking data. We propose methodology to model specific continuous responses with a functional linear model utilizing spectra obtained from the local fast Fourier transform (FFT) of walking as a predictor. Utilizing prior knowledge of the mechanics of walking, we incorporate this as additional information for the structure of our transformed walking spectra. The methods were applied to the in-the-laboratory data obtained from the Developmental Epidemiologic Cohort Study (DECOS).

## 1. Introduction

Use of wearable accelerometers has become increasingly common in studies of physical activity, aging, and obesity [1,2,3,4,5,6,7,8]. Self-reported measures, such as questionnaires, have been widely used to assess physical activity (PA) previously [6,9]. One reason why we care about using accelerometry over self-reporting is because there are some populations whose self-reported measures can be inaccurate [10]. One such population is older adults. Questionnaires require individuals to recall their daily activities, which can be extremely difficult, particularly for older individuals [11]. Schrack et al. [12] showed there are changes in the daily patterns and amount of PA as people age. Accelerometry is also an important tool that can be applied to the general population, because walking is the most popular form of aerobic physical activity [13].

Accelerometers offer a non-invasive and objective alternative to self-reporting methods. Advancements in data processing allow for the analysis of specific gait characteristics, such as cadence and asymmetry [14]. Likewise, advancements in statistical methodology for analysis of high dimensional data have opened up new paths for analyzing more complex and potentially more informative summaries of accelerometry data. Accelerometers are electro-mechanical devices that measure acceleration along three orthogonal axes. They are often worn on a person’s waist or wrist, and they provide high frequency, high-throughput data represented by three time series of acceleration measurements [15,16]. The data are typically collected at the sub-second level (usually between 10 to 100 observations per second); however, most studies aggregate the data over one minute epochs, or windows, and often, using a user specified threshold, the data are characterized into activity counts per minute. While thresholding methods are useful in describing the timing and duration for certain levels of PA, many nuances of the data are lost. For example, it is not possible to evaluate how a person is walking from activity count summaries. Especially in certain populations, such as older or obese populations, this raises the question as to whether a more detailed quantification of the walking signal can provide additional information. For example, if an older person’s legs are bothering them, we may detect signs of limping that could help explain the low levels of PA. Our strategy is to extract detailed information from the raw accelerometry signal during periods of walking. We transform this information into useful quantities, and then we build regression models to associate characteristics of walking with continuous responses.

**Figure 1:**
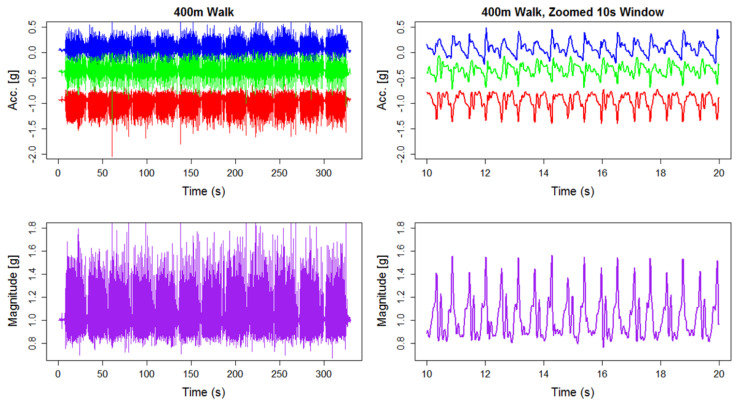
Triaxial accelerometer data from the 400 m walk for a single individual (**top left**) and a zoomed 10 s window (**top right**). Vector magnitude from the 400 m walk for same individual (**bottom left**) and zoomed 10 s window (**bottom right**).

To illustrate the complex nature of the data collected, Figure 1 shows the raw data collected from a triaxial accelerometer (Actigraph GT3X+) for a single individual during an in-laboratory 400 m walk. The top left panel of Figure 1 presents the entire 400 m walk, where each axis is shown in a different color. With just over 5 min worth of data, the characteristics of the signal are nearly impossible to visualize. The top right panel of Figure 1 shows a 10 s window of the same data. At this scale, we can discern a fairly periodic signal. In the bottom row of Figure 1, we present the vector magnitude of the same data presented in the top row. We can see that the periodic nature of the data are preserved while information about the three dimensional direction is lost. However, in free-living data collection, it is difficult to control the orientation of the device when participants are able to remove the device. For this reason, the magnitude of the signal is sufficient and relatively stable to capture the gait characteristics described in this manuscript. The periodic characteristics of walking naturally lend themselves to a frequency analysis approach for quantifying the features of walking. By utilizing the methods described in Urbanek et al. [15], we can extract estimates of cadence (steps per second) and average magnitude from windows of raw data. In addition to these more common features, we also utilize the spectra obtained from the local fast Fourier Transform (FFT) as a functional predictor for modeling the association of walking with continuous responses such as age and body mass index (BMI). By incorporating the walking spectra as a predictor, we gain additional information about the characteristics of a person’s gait which may be associated with the response of interest. For example, an individual with a very smooth walking stride would result in most of the energy from walking concentrated around the frequencies near the cadence. However, an individual with an interrupted stride (e.g., a limp) would result in energy being more dispersed through higher frequencies.

Several methods exist for fitting a scalar-on-regression function, such as $y=\int_{I}W\left(s\right)\beta \left(s\right)ds+\;\epsilon$, where W(·) is a functional predictor and *y* is a scalar response variable. Several methods for estimating β(·) are based on the eigenfunctions associated with some covariance operator defined by the predictors [17]. Due to the periodic nature of walking, we have strong reason to believe the vast majority of information contained in the walking spectra will be located around the harmonics centered at multiples of the dominant frequency. The PEER method developed by Randolph et al. [17] allows for the incorporation of presumed structure directly into the estimation process and is preferable to a purely empirical estimator. This particular method has been widely used in other areas of research, for example, in heritability and evolution studies [18,19] and in microbiome analysis [20]. However, to the best of our knowledge, this is a novel use of the PEER method in the analysis of the raw accelerometry data.

In this manuscript, we propose a novel application of recently developed statistical methods for the analysis of accelerometry data by associating continuous responses, such as age and BMI, with the Fourier spectrum of walking. The purpose of this manuscript is to serve as a proof of concept for researchers seeking to utilize more information from the accelerometry data in modeling associations between characteristics of walking and health related outcomes. We show how this additional information can be combined with scalar predictors in a linear regression model framework. The remainder of this manuscript is structured as follows. In Section 2, we describe the data collection and pre-processing procedures. In Section 3, we describe the functional linear model used to fit the data and how the estimation is performed. In Section 4, we apply the proposed model to data collected in the laboratory from a study of an aging adult population. In Section 5, we conclude with a discussion.

## 2. Data Collection and Pre-Processing

Eighty-nine community-dwelling older adults were recruited from the Pittsburgh, Pennsylvania area for the National Institute on Aging, Aging Research Evaluating Accelerometry (AREA) project, part of the Developmental Epidemiologic Cohort Study (DECOS) [3]. AREA was a cross-sectional methodological initiative designed to examine the impact of accelerometry wear location on assessment of physical activity and sedentary behavior among 89 older adults enrolled between March and May of 2010 [21]. The report included data from 51 healthy participants (25 men and 26 women) who had complete the “in-the-lab” (N=46) or “in-the-wild” (N=48) accelerometry data. Individuals were excluded from the DECOS study for the following reasons: hip fracture, stroke in the past 12 months, cerebral hemorrhage in the past 6 months, heart attack, angioplasty, heart surgery in the past 3 months, chest pain during walking the past 30 days, current treatment for shortness of breath or a lung condition, usual aching, stiffness, or pain in their lower limbs and joints, and bilateral difficulty in bending or straightening the knees fully [22]. This paper focuses on the N=46 participants that completed the “in-the-lab” fast-paced 400 m walk. Data were collected with an Actigraph GT3X+ worn at the right hip. The devices collected raw accelerometry data along three orthogonal axes at a sampling frequency of 80 Hz. A summary of the demographic data for all N=46 participants is provided in Table 1.

The first step in pre-processing the data is to split the observed triaxial signal from the 400 m walk into 10 s non-overlapping windows. For each window, we transform the raw triaxial signal into vector magnitude (VM), where VM is defined as the root sum of squares of the three axes, i.e., $vm\left(t\right)=\sqrt{x_{1}{\left(t\right)}^{2}+x_{2}{\left(t\right)}^{2}+x_{3}{\left(t\right)}^{2}}$. The vector magnitude count (VMC) is then calculated as the mean absolute deviation of the VM:

(1) $$v_{\tau }\left(t\right)=\frac{1}{\tau }\sum_{u=t-\tau /2}^{t+\tau /2}\left|vm\left(u\right)-\bar{vm}\right|,$$

where τ is the window size expressed as number of sampling points [15]. We then transform the VM from the time domain into the frequency domain using the local FFT, or short time Fourier transform (STFT). Similarly to Urbanek et al. [15], we define the STFT at time *t* of the vm(t) as

(2) $$X\left(t,f;\tau \right)=\sum_{u=[t-\tau /2]}^{\left[t+\tau /2\right]}vm\left(u\right)h\left(u\right)e^{-i2\pi fu/\tau },$$

where *f* is the frequency index and τ is a tuning parameter specifying the number of observations in the interval centered at *t*. The Hanning weights, defined as, h(u;τ)=0.5[1−cos{2πu/(τ−1)}] are used to avoid a blurring of the obtained spectra which can happen as a result of the windowing process. The spectrum is then defined as the absolute value of the STFT, |X(t,f;τ)|.

**Figure 2:**
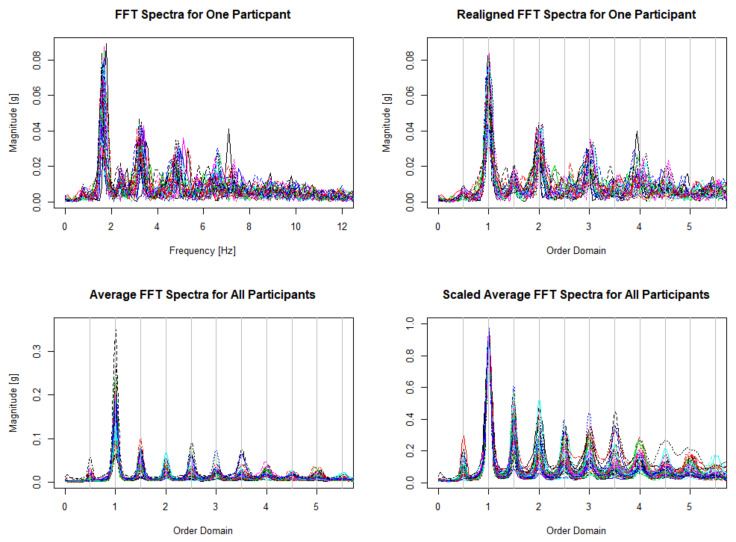
Pre-processing data. Observed FFT spectra for one participant as described in step 4 of Algorithm 1 (**top left**). Observed spectra realigned into order domain for the same participant as described in step 6 of Algorithm 1 (**top right**). Average realigned spectra for all participants as described in step 7 of Algorithm 1 (**bottom left**). Scaled average spectra for all participants as described in step 9 of Algorithm 1 (**bottom right**).

For each spectrum obtained, we then identify the fundamental frequency (cadence) as the location of the largest peak in the spectrum. Since the reported frequency of walking is between 1.4 and 2.5 Hz [23], which corresponds to 1.4 to 2.5 steps per second, we look for the cadence in a conservative range of 1.2–4.0 Hz to be consistent with Urbanek et al. [15]. The frequency axis used is from 0 to 39.9 Hz sampled every 0.1 Hz which ensures every individual’s spectra will contain at least 10 multiples of their dominant frequency, or cadence. In the top left panel of Figure 2, we display all spectra for a single participant. Although these spectra appear similar, it is evident that there is variability between spectra obtained in different windows. Therefore, once the spectra are all obtained, and we have identified their fundamental frequencies, it is imperative that we align each spectrum for aggregation.

In order to align all spectra at their fundamental frequency, we further transform each spectrum from the frequency domain into the order domain by scaling the frequency axis by the fundamental frequency for each spectrum. Linear interpolation is then used to place each spectrum back on the same sampling grid. This ensures that all spectra are aligned and sampled at equally spaced points. The top right panel of Figure 2 illustrates how the realigned spectra for a single participant. As can be seen, all spectra are perfectly aligned at the dominant frequency.

However, each spectrum is sampled discretely, therefore, further harmonics may be slightly misaligned in the order domain. To compensate, we average the spectra across all windows for each participant in order to obtain a global estimate of walking features for each individual. Each spectrum is restricted to 546 points between 0.3 and 5.75 in the order domain to avoid modeling signal noise at the beginning and end of the spectra. The bottom left panel of Figure 2 shows the averaged spectra for all N=46 participants. The peaks of the average spectra for each individual are now nearly perfectly aligned in the order domain at multiples of the fundamental frequency.

Finally, we scale each individual’s average spectrum by the magnitude of the spectrum at the cadence. By scaling the spectra in this way, the magnitude at each harmonic can be interpreted as a ratio to the magnitude at the cadence. This process is illustrated in bottom right panel of Figure 2, and this entire process is fully detailed in Algorithm 1. These steps for pre-processing the raw accelerometry data are essential in order to properly fit the statistical model described in Section 3.
**Algorithm 1:** Steps for pre-processing the raw accelerometry data. **Input**: x(t)—accelerometry signal, $f_{s}$—sampling frequency, $s_{min}=1.2$ Hz, $s_{max}=4.0$ Hz  **Output**: FFT—scaled average FFT spectrum, VMC—average VMC, Cadence—average cadence 
1 Divide accelerometry signal into 10 s non-overlapping windows. 
2 Transform accelerometry signal into vector magnitude vm(t). 
3 Compute vector magnitude count, $v_{10}\left(t\right)$, for each window. 
4 Compute Fourier spectrum for each window. 
5 Estimate cadence as frequency centered under the largest peak in spectrum. 
6 Transform spectra from frequency domain to order domain by scaling frequency axis by the frequency of the cadence. 
7 Average vector magnitude count, cadence, and order domain spectra across all windows. 
8 Restrict spectra to points between 0.3 and 5.75 multiples of the cadence frequency. 
9 Scale spectra by magnitude of the average spectra at the cadence.

## 3. Statistical Methods

Frequently, the methods applied to analyze the data arising from the raw accelerometry signal rely on the discrete features extracted from such data, leading to possible loss of information. In contrast, we take the full time series signal into account and work with its continuous properties, namely, the spectrum obtained from the walking portion of the accelerometry signal. We summarize the approach taken below.

Let $W_{i}\left(\cdot \right)$ denote a functional predictor (e.g., a scaled average FFT spectrum) from the *i*th study participant where (i=1,…,N). We will assume that each observed predictor is obtained as a discretized version of an idealized function at *p* equally-spaced points, $s_{1},\ldots ,s_{p}$, as can be seen in the walking spectra in Figure 2. We let $w_{i}:={\left[w_{i}\left(s_{1}\right),\ldots ,w_{i}\left(s_{p}\right)\right]}^{T}$ be the p×1 vector of values sampled from $W_{i}\left(\cdot \right)$. Then our observed data take the form $\left\{y_{i};x_{i};w_{i}\right\}$ where $y_{i}$ is a scalar response, $x_{i}$ is a K×1 vector of measurements from *K* scalar predictors (e.g., sex or average cadence), and $w_{i}$ is the functional predictor from the *i*th participant. We denote the true coefficient function by β(·), and then, the functional regression model of interest is given by

(3) $$y_{i}=x_{i}^{T}\gamma +\int_{I}W_{i}\left(s\right)\beta \left(s\right)ds+\epsilon_{i}$$

where $\epsilon_{i}∼N\left(0,\sigma_{\epsilon }^{2}\right)$. Here $x_{i}^{T}\gamma$ is the linear effect from *K* scalar predictors and $\int_{I}W_{i}\left(s\right)\beta \left(s\right)ds$ is the functional effect.

### 3.1. Estimation of Parameters

Several approaches can be used to estimate the association between the scalar $x_{i}$ and functional $w_{i}\left(\cdot \right)$ predictors with the outcome $y_{i}$. In our work, we utilize the approach proposed in Randolph et al. [17], which incorporates functional structure into the estimation of β(·). Specifically, the properties of the estimated spectra, i.e., their continuity, smoothness, and common behavior, are taken into the estimation procedure explicitly. To represent our model in a compact form, we combine the data from all *N* participants and express Equation (3) as

(4) y=Xγ+Wβ+ϵ

where $y={\left[y_{1},\ldots ,y_{N}\right]}^{T}$ is an N×1 vector of responses; $X={\left[x_{1}^{T},\ldots ,x_{N}^{T}\right]}^{T}$ is an N×K design matrix corresponding to the scalar predictors with coefficient vector γ; $W={\left[w_{1}^{T},\ldots ,w_{N}^{T}\right]}^{T}$ is an N×p design matrix corresponding to the functional predictors with functional coefficient vector β.

**Figure 3:**
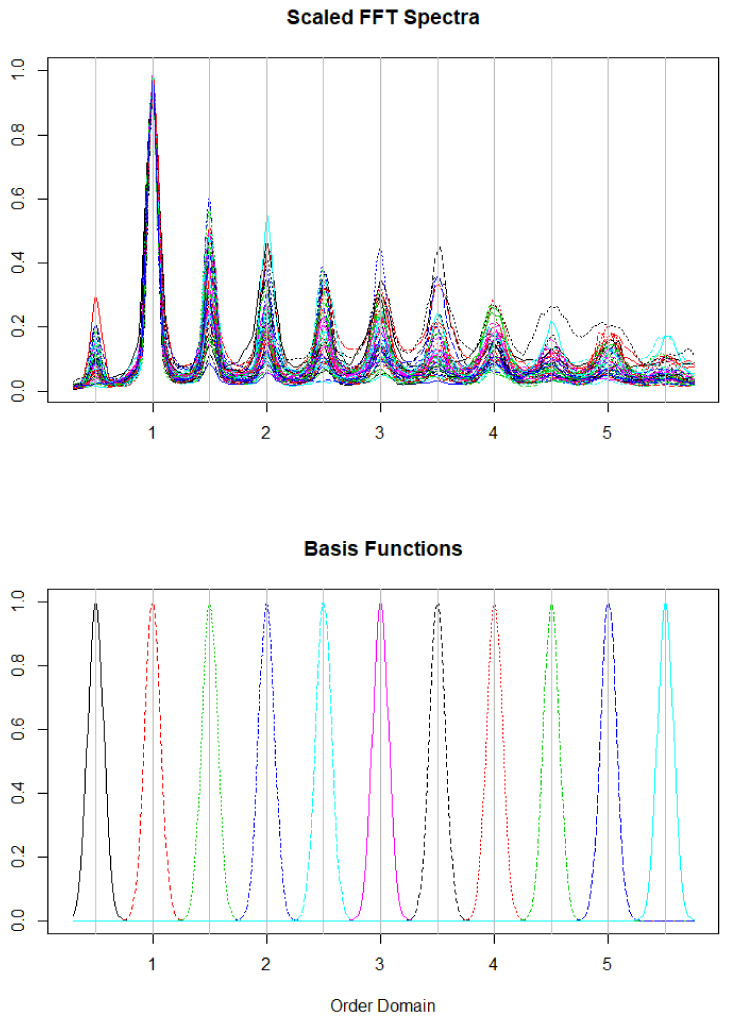
Pre-processed walking spectra (**top**) and basis functions used for modeling (**bottom**). The *x*-axis represents multiples of the frequency of the cadence.

Given the periodic nature of the walking behavior, if the walking spectral properties are associated with the outcomes, the relevant information contained in the walking spectra is localized around the harmonics at multiples of the dominant frequency. We thus want to estimate β while imposing this prior information on its functional structure. We achieve this by using the penalty operator, *L* [17], which is created from the basis functions in the right panel of Figure 3. The penalized estimates of γ and β are obtained as the solution to the following criterion

(5) $${\left[\tilde{\gamma },{\tilde{\beta }}_{\lambda ,L}\right]}^{T}=\underset{\gamma ,\beta }{arg min}\{||y-X\gamma -{W\beta ||}^{2}+{\lambda ||L\beta ||}_{L^{2}}^{2}\},$$

where we only penalize the functional coefficient vector β.

Given some prior knowledge about the structure of our functional predictor, the penalty is defined utilizing a subspace containing this information [17]. We define this informative space, *Q*, to be a span of basis functions (right panel of Figure 3) emphasizing the relevant features of β(·) and consider the orthogonal projection $P_{Q}=QQ^{+}$. As described in Randolph et al. [17], we define the decomposition-based penalty as

(6) $$L\equiv L_{Q}=a\left(I-P_{Q}\right)+P_{Q}$$

for some a>0. When a>1 the estimate is penalized more in the non-informative space orthogonal to *Q*. When a=1, the estimate is simply an ordinary ridge regression estimate. Therefore, a generalized ridge estimate of γ and β can be obtained as

(7) $${\left[\tilde{\gamma },\tilde{\beta }\right]}^{T}={\left(X_{o}^{T}X_{o}+\lambda L_{o}^{T}L_{o}\right)}^{-1}X_{o}^{T}y,$$

where $X_{o}=\left[X\;W\right]$ and $L_{o}=\mathrm{blockdiag}\left\{0,L_{Q}^{T}L_{Q}\right\}$. The tuning parameter, λ, is estimated in a principled way via a linear mixed model equivalence, as described in Ruppert et al. [24]. Specifically, the optimization criterion (5) is written in an equivalent linear mixed model form with the coefficients γ being fixed and the coefficients β being random with a distribution $\beta ∼N(0,\sigma_{\beta }^{2})$. The estimate of the tuning parameter, λ, is then simply the ratio of the variances $\sigma_{\epsilon }^{2}$ and $\sigma_{\beta }^{2}$.

## 4. DECOS Example

We applied the methods discussed in Section 3 to the data described in Section 2 to study the associations of walking spectra obtained from the fast-paced 400 m walk with age and BMI [3]. The fast-paced 400 m walk is often used in epidemiological studies of older adults to assess aerobic fitness [25]. The most common protocol implemented for the fast-paced 400 m walk is the long distance corridor walk (LDCW) [26]. The pre-processed walking spectra described in Section Figure 2 were each sampled at k=546 distinct sampling points within 0.3 and 5.75 of the order domain. This range was chosen because there is little energy contained in the spectra beyond 13.5 Hz. Assuming an average cadence of 2.0 Hz, this range sufficiently covers the relevant features of walking. There were N=46 participants that completed the LDCW. In addition to each participant’s scaled average walking spectra, an estimate of their average cadence was used as a predictor in the proposed models to control for participant-specific walking speeds. In addition, VMC was used to control for the energy magnitude each participant produced. For example, an individual with a very controlled and smooth walking style would have shown lower magnitude than an individual with a heavy stomp in their walk. We also adjusted each model for gender differences.

In order to use our prior knowledge about the structure of the walking spectra, we define a penalty $L_{Q}$ as given in Equation (6) (with a=2). We define our basis functions as normal density functions centered at multiples of the cadence from 0.5×cadence to 5.5×cadence using steps of 0.5×cadence. We chose a standard deviation such that the distributions were nearly orthogonal. Scaled average walking spectra and basis functions are displayed in Figure 3.

Following the general formulation of the functional regression model (3), we fit the following model to these data:

(8) $$y_{i}=\gamma_{0}+{Male}_{i}*\gamma_{1}+{Cadence}_{i}*\gamma_{2}+{VMC}_{i}*\gamma_{3}+\int_{I}{Spectrum}_{i}\left(s\right)\beta \left(s\right)ds+\epsilon_{i}$$

where $y_{i}$ is either age or BMI, ${Male}_{i}$ is a binary variable, and ${Cadence}_{i}$ and ${VMC}_{i}$ are the cadence and vector magnitude count for participant *i*, respectively. ${Spectrum}_{i}\left(\cdot \right)$ is the scaled average walking spectrum for participant *i* as described in Section 2. We assume that $\epsilon_{i}∼N\left(0,\sigma_{\epsilon }^{2}\right)$. Regression coefficients γ and the regression function β(s) are estimated via the procedure described in Section 3.1 using the peer() function from the refund package in R [27,28]. Thus, the scalar outcome $y_{i}$ is predicted via a weighted sum of the products of the collected data (indicator variable of male sex, cadence, VMC, and a spectrum) and their respective estimated regression coefficients $\tilde{\gamma }$’s and $\tilde{\beta }\left(\cdot \right)$.

**Figure 4:**
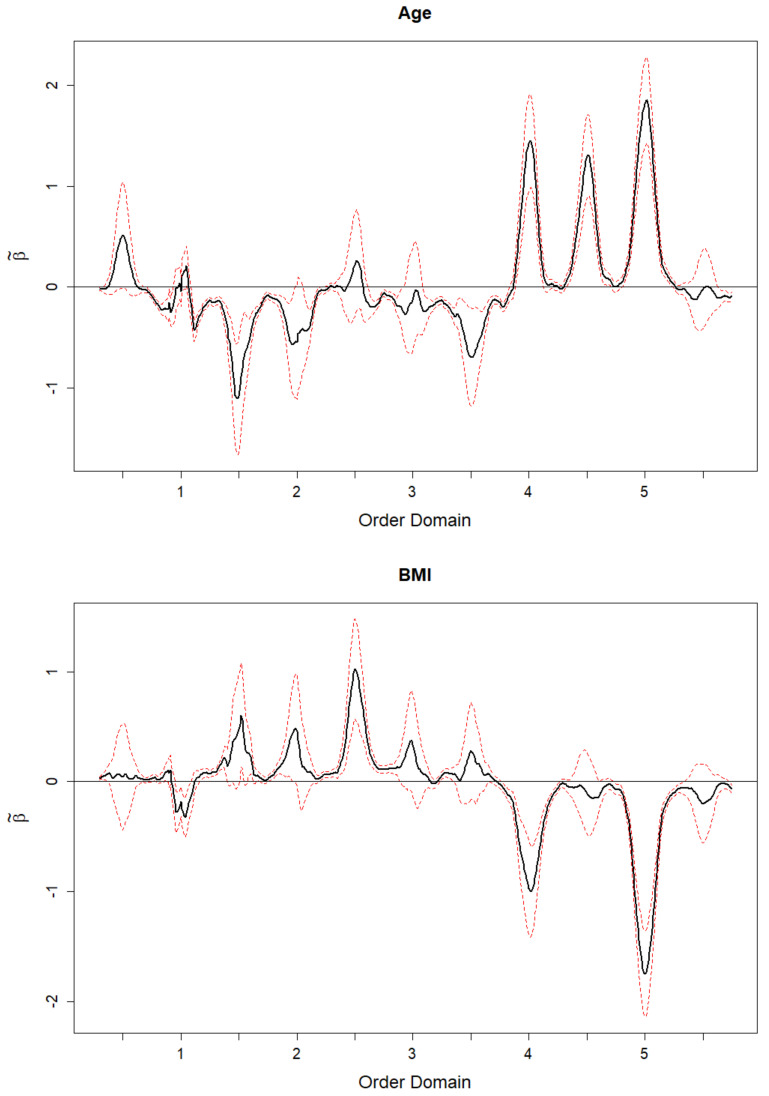
Estimates of the coefficient function, $\tilde{\beta }$, (with 95% point-wise confidence band) for the association of walking with age and BMI, as described in Section 4. The x-axis represents multiples of the frequency of the cadence.

Figure 4 displays the estimates of β(·) along with the pointwise 95% confidence bands for the two models described in Equation (8). These figures show that the estimated regression function is different from zero at different multiples of the cadence. The regression function for age (top) shows that the coefficient function, $\tilde{\beta }$, is negative at the multiples 1.5 and 3.5 and positive at the multiples 4, 4.5, and 5, whereas for the other multiples the estimated coefficients are not significantly different from zero. These results indicate that younger individuals have larger magnitude in the lower harmonics relative to the magnitude at their cadence which indicates a heavier stomp component and controlled walking motion. Older individuals have larger magnitudes in the higher harmonics relative to the magnitudes of their cadence, which indicates a less controlled compensatory walking motion (e.g., a limp). These results seem to be consistent with findings of prior research [29,30,31,32,33,34,35]. However additional research is needed to validate our conclusions.

The regression results for BMI (bottom) show that the coefficient function, $\tilde{\beta }$, is positive at the multiple 2.5 and negative at the multiples 4 and 5, whereas for the other multiples the estimated coefficients are not significantly different from zero. These results could indicate that overweight or obese individuals tend to walk with a heavier stomp component, resulting in higher magnitudes of the lower harmonics than the normal weight individuals. Leaner individuals walk with a lighter stomp component, resulting in walking characteristics with lower magnitudes in the lower harmonics and higher magnitudes in the higher harmonics. Further discussion and possible limitations of this interpretation are provided in Section 5.

Both results described above show that the information provided by the penalty reduces the number of spurious findings and at the same time emphasizes the signal content of the scaled Fourier spectra.

## 5. Discussion

In this paper we proposed a novel application of existing functional linear model methods to the study of physical activity data collected by accelerometers. We proposed an algorithm for pre-processing the raw data collected from accelerometers to quantify the characteristics of walking in a more detailed manner than is typically used with activity count summaries. By utilizing the periodic characteristics of walking, we were able to reduce the dimensionality of the raw data into a form that retained some details of the original signal while allowing us to use existing statistical methods for analyses. We applied these methods to the in-the-laboratory data collected from a study of an older adult population.

While FFT has been widely used for pre-processing accelerometry data, the features extracted from such methods have been applied to the problem of classification of activity types as opposed to associating characteristics of walking to continuous response variables [15,36,37,38]. To our knowledge, this is the first proposed application of functional linear regression techniques to model the association of walking spectra with continuous responses. Due to the periodic characteristics of walking, the proposed method naturally lends itself to this application, wherein we can inform the penalty operator of where the relevant information is contained in the spectra. This method is not limited to the cross-sectional setting, as demonstrated in this paper, and it is easily extended to responses collected longitudinally [39]. In addition to walking speed, this more detailed quantification of walking may provide additional information as to how certain degenerative diseases (e.g., Parkinson’s disease and multiple sclerosis) affect a person’s ability to walk over the progression of disease. Reuter et al. [40] showed that certain walking programs can actually improve gait characteristics of individuals with Parkinson’s disease over the course of a 6-month study. Gait characteristics were measured on a specialized treadmill outfitted with specialized sensors to accurately measure foot-ground contact. The application of these proposed methods could alleviate any financial restrictions of such studies to allow for much larger randomized prospective studies to determine whether these exercise therapies actually slow down the progression of such diseases. Utility of these methods can only be assessed with the inclusion of accelerometers in such studies, and they are being increasingly used.

We acknowledge that there are limitations in our analyses. For example, we did not collect data from the 3D motion capture or ground force reaction (GFR) measurements to validate the findings of our analyses. This manuscript is meant to demonstrate how researchers can utilize existing statistical methodology to analyze finer features of the raw accelerometry data retaining additional information about characteristics and changes in gait that are usually lost due to over-aggregation of the raw data collected. It was beyond the scope of this study to obtain information from specialized laboratory equipment, but we do feel that further research is warranted to validate our findings and draw associations between the additional characteristics of gait observed in our analyses with gait measurements obtained in a laboratory. Ko et al. [33] found that older adults with obesity modify their gait patterns compared to normal weight counterparts while walking at normal and fast speeds. For example, they found that obese participants had lower mechanical work expenditure (MWE) in the ankle and significantly higher MWE in the knee and hip compared to normal weight participants. Paired with the raw accelerometry data, these findings could identify the specific mechanisms validating the additional associations we found in our analyses. Menz et al. [31] observed that older participants exhibited a more conservative gait pattern characterized by reduced velocity, shorter step length, and increased step timing variability which could be contributing to the portion of the signals observed at the higher frequencies of the walking spectra in our study.

In conclusion, we acknowledge that age and BMI are easy to measure, but the strength of this study is that it serves as a proof of concept for how researchers can utilize the extracted walking characteristics in the presence of more relevant health related outcomes, e.g., fatigability or neurocognitive function. In addition, we studied only relatively healthy elderly individuals. Thus, generalizability of the findings to either healthy younger individuals or unhealthy older individuals needs to be studied. Given the small sample size of our study and utilization of the data from the laboratory experiment only, additional research is needed to establish similar associations between the health outcomes and the free-living walking data characteristics.

## Figures and Tables

**Table 1:** DECOS participant characteristics (N=46).

Characteristic	Summary Statistics
Male (n (%))	22 (47.8%)
Age (Mean (SD))	78.24 (5.74)
BMI (Mean (SD))	26.73 (3.94)
Cadence (Mean (SD))	2.06 (0.17)
VMC (Mean (SD))	0.25 (0.09)

## References

[1] Cooper R., Huang L., Hardy R., Crainiceanu A., Harris T., Schrack J.A., Crainiceanu C., Kuh D. (2017). Obesity history and daily patterns of physical activity at age 60–64 years: Findings from the MRC National Survey of Health and Development. J. Gerontol. Ser. Biomed. Sci. Med. Sci..

[2] Keane E., Li X., Harrington J.M., Fitzgerald A.P., Perry I.J., Kearney P.M. (2017). Physical activity, sedentary behavior and the risk of overweight and obesity in school-aged children. Pediatr. Exerc. Sci..

[3] Lange-Maia B.S., Newman A.B., Strotmeyer E.S., Harris T.B., Caserotti P., Glynn N.W. (2015). Performance on fast-and usual-paced 400-m walk tests in older adults: are they comparable?. Aging Clin. Exp. Res..

[4] Healy G.N., Winkler E.A., Brakenridge C.L., Reeves M.M., Eakin E.G. (2015). Accelerometer-derived sedentary and physical activity time in overweight/obese adults with type 2 diabetes: Cross-sectional associations with cardiometabolic biomarkers. PLoS ONE.

[5] Copeland J.L., Esliger D.W. (2009). Accelerometer assessment of physical activity in active, healthy older adults. J. Aging Phys. Act..

[6] Pruitt L.A., Glynn N.W., King A.C., Guralnik J.M., Aiken E.K., Miller G., Haskell W.L. (2008). Use of accelerometry to measure physical activity in older adults at risk for mobility disability. J. Aging Phys. Act..

[7] Gardner A.W., Poehlman E.T. (1998). Assessment of free-living daily physical activity in older claudicants: Validation against the doubly labeled water technique. J. Gerontol. Ser. Biol. Sci. Med. Sci..

[8] Richardson C.A., Glynn N.W., Ferrucci L.G., Mackey D.C. (2015). Walking energetics, fatigability, and fatigue in older adults: The study of energy and aging pilot. J. Gerontol. Ser. Biol. Sci. Med. Sci..

[9] Losina E., Yang H., Stanley E., Katz J., Collins J. (2019). Physical activity as a novel outcome of total knee replacement: Comparing self-report and objective PA assessments. Osteoarthr. Cartil..

[10] Sabia S., Cogranne P., van Hees V.T., Bell J.A., Elbaz A., Kivimaki M., Singh-Manoux A. (2015). Physical activity and adiposity markers at older ages: Accelerometer vs questionnaire data. J. Am. Med. Dir. Assoc..

[11] Baranowski T. (1988). Validity and reliability of self report measures of physical activity: An information-processing perspective. Res. Q. Exerc. Sport.

[12] Schrack J.A., Zipunnikov V., Goldsmith J., Bai J., Simonsick E.M., Crainiceanu C., Ferrucci L. (2014). Assessing the “physical cliff”: Detailed quantification of age-related differences in daily patterns of physical activity. J. Gerontol. Ser. Biol. Sci. Med. Sci..

[13] Centers for Disease Control and Prevention (2013). More People Walk to Better Health. https://www.cdc.gov/vitalsigns/Walking/.

[14] Moe-Nilssen R., Helbostad J.L. (2004). Estimation of gait cycle characteristics by trunk accelerometry. J. Biomech..

[15] Urbanek J.K., Zipunnikov V., Harris T., Fadel W., Glynn N., Koster A., Caserotti P., Crainiceanu C., Harezlak J. (2018). Prediction of sustained harmonic walking in the free-living environment using raw accelerometry data. Physiol. Meas..

[16] Sandroff B.M., Motl R.W., Suh Y. (2012). Accelerometer output and its association with energy expenditure in persons with multiple sclerosis. J. Rehabil. Res. Dev..

[17] Randolph T.W., Harezlak J., Feng Z. (2012). Structured penalties for functional linear models–partially empirical eigenvectors for regression. Electron. J. Stat..

[18] Kulbaba M.W., Clocher I.C., Harder L.D. (2017). Inflorescence characteristics as function-valued traits: Analysis of heritability and selection on architectural effects. J. Syst. Evol..

[19] Gomulkiewicz R., Kingsolver J.G., Carter P.A., Heckman N. (2018). Variation and evolution of function-valued traits. Annu. Rev. Ecol. Evol. Syst..

[20] Randolph T.W., Zhao S., Copeland W., Hullar M., Shojaie A. (2018). Kernel-penalized regression for analysis of microbiome data. Ann. Appl. Stat..

[21] Urbanek J.K., Zipunnikov V., Harris T., Crainiceanu C., Harezlak J., Glynn N.W. (2017). Validation of gait characteristics extracted from raw accelerometry during walking against measures of physical function, mobility, fatigability, and fitness. J. Gerontol. Ser. A.

[22] Lange-Maia B.S., Strotmeyer E.S., Harris T.B., Glynn N.W., Simonsick E.M., Brach J.S., Cauley J.A., Richey P.A., Schwartz A.V., Newman A.B. (2015). Physical activity and change in long distance corridor walk performance in the health, aging, and body composition study. J. Am. Geriatr. Soc..

[23] Pachi A., Ji T. (2005). Frequency and velocity of people walking. Struct. Eng..

[24] Ruppert D., Wand M.P., Carroll R.J. (2003). Semiparametric Regression.

[25] Simonsick E.M., Fan E., Fleg J.L. (2006). Estimating cardiorespiratory fitness in well-functioning older adults: Treadmill validation of the long distance corridor walk. J. Am. Geriatr. Soc..

[26] Simonsick E.M., Montgomery P.S., Newman A.B., Bauer D.C., Harris T. (2001). Measuring fitness in healthy older adults: The Health ABC Long Distance Corridor Walk. J. Am. Geriatr. Soc..

[27] Goldsmith J., Scheipl F., Huang L., Wrobel J., Gellar J., Harezlak J., McLean M.W., Swihart B., Xiao L., Crainiceanu C. (2020). Refund: Regression with Functional Data; R Package Version 0.1-22. https://CRAN.R-project.org/package=refund.

[28] R Core Team (2019). R: A Language and Environment for Statistical Computing.

[29] Gabell A., Nayak U. (1984). The effect of age on variability in gait. J. Gerontol..

[30] Öberg T., Karsznia A., Öberg K. (1993). Basic gait parameters: Reference data for normal subjects, 10–79 years of age. J. Rehabil. Res. Dev..

[31] Menz H.B., Lord S.R., Fitzpatrick R.C. (2003). Age-related differences in walking stability. Age Ageing.

[32] Kavanagh J.J., Menz H.B. (2008). Accelerometry: A technique for quantifying movement patterns during walking. Gait Posture.

[33] Ko S.u., Stenholm S., Ferrucci L. (2010). Characteristic gait patterns in older adults with obesity—Results from the Baltimore Longitudinal Study of Aging. J. Biomech..

[34] Harding G.T., Hubley-Kozey C.L., Dunbar M.J., Stanish W.D., Wilson J.L.A. (2012). Body mass index affects knee joint mechanics during gait differently with and without moderate knee osteoarthritis. Osteoarthr. Cartil..

[35] Moissenet F., Leboeuf F., Armand S. (2019). Lower limb sagittal gait kinematics can be predicted based on walking speed, gender, age and BMI. Sci. Rep..

[36] Zhang S., Rowlands A.V., Murray P., Hurst T.L. (2012). Physical activity classification using the GENEA wrist-worn accelerometer. Med. Sci. Sports Exerc..

[37] Preece S.J., Goulermas J.Y., Kenney L.P., Howard D. (2009). A comparison of feature extraction methods for the classification of dynamic activities from accelerometer data. IEEE Trans. Biomed. Eng..

[38] Mannini A., Intille S.S., Rosenberger M., Sabatini A.M., Haskell W. (2013). Activity recognition using a single accelerometer placed at the wrist or ankle. Med. Sci. Sports Exerc..

[39] Kundu M.G., Harezlak J., Randolph T.W. (2016). Longitudinal functional models with structured penalties. Stat. Model..

[40] Reuter I., Mehnert S., Leone P., Kaps M., Oechsner M., Engelhardt M. (2011). Effects of a flexibility and relaxation programme, walking, and nordic walking on Parkinson’s disease. J. Aging Res..

